# Social Media, Health Consciousness, and Cultural Influences on Sugar Reduction Behaviors in Chinese Youth: Extending the Stimulus-Organism-Response Model

**DOI:** 10.2196/68180

**Published:** 2025-12-19

**Authors:** Bing Hu, Yi Zhu, Ruxiang Bao, Ziying Zhao, Chao Liu, Aomi Lin

**Affiliations:** 1 College of Journalism and Communication, Huaqiao University, No. 668, Jimei Avenue, Xiamen, Fujian, 361021, China, 86 15759213683; 2 School of Economics and Finance, Huaqiao University, Quanzhou, Fujian, China; 3 Business Analytics Research Center, Chang Gung University, Xiamen, Taoyuan, Taiwan

**Keywords:** social media usage, sugar reduction behaviors, conformity, health consciousness, eHealth literacy, health promotion, social media, SOR model, cultural influences, sugar reduction, adolescent, health behavior, sugar consumption, stimulus-organism-response

## Abstract

**Background:**

The rising prevalence of sugar-related diseases, such as obesity and diabetes, has intensified efforts to reduce sugar intake, particularly among youth. In China, social media is playing an increasingly significant role in shaping health behaviors, including habits related to sugar consumption, as sugar reduction has become a prominent youth-led movement.

**Objective:**

This study extends the stimulus-organism-response (SOR) model by incorporating the distinct cultural influence of “face” to investigate the impact of social media on sugar reduction behaviors (SRBs) among Chinese youth, as well as the mediating role of health consciousness (HC) and conformity, and the moderating effects of face concern (FC) and eHealth literacy (EHL).

**Methods:**

We conducted a national web-based, cross-sectional survey through proportionate probability sampling of 883 Chinese youth in July 2024. Descriptive statistics, Pearson correlations, model fit indices, and partial least squares. Structural equation models were used to examine the relationships among all variables.

**Results:**

Nearly half of the 883 participants were female (460/883, 52.1%), 91.9% (812/883) were aged 15‐30 years. Most participants (602/883, 68.2%) had undergraduate education levels; the majority (688/883, 77.9%) had a bachelor’s degree or higher, and 654 (74.1%) had a normal BMI. Most participants (575/883, 74.1%) had used social media for 3‐10 years. Chinese youth reported relatively high SRB scores (mean 3.62, SD 0.99). Male participants achieved notably higher scores (mean 3.72, SD 0.93), whereas participants aged 15‐18 years showed significantly lower SRB scores (mean 3.50, SD 1.05). Structural equation modeling revealed that social media usage positively influenced conformity (β=.51; *P*<.001) and HC (β=.35; *P*<.001). These factors, in turn, significantly predicted SRBs (β=.14 and β=.50, respectively; both *P*<.001). The influence of social media usage on SRBs is primarily facilitated through 2 mediating pathways: HC mediated the relationship (Variance Accounted For=51.5%), while conformity’s mediation was less pronounced (Variance Accounted For=21.05%), indicating a secondary influence. FC (β=.09; *P*=.02) and EHL (β=.06; *P*=.04) moderated the respective relationships.

**Conclusions:**

This study demonstrates that social media effectively promotes SRBs among Chinese youth. By embedding cultural influences, such as FC, alongside enabling competencies, such as EHL, in an extended SOR model, we enhance our understanding of social media’s influence on health behaviors. The findings highlight cultural nuances in health communication and position the enhanced SOR model as a framework for health promotion. Furthermore, the study underscores the primary mediating effect of HC—surpassing that of conformity—while also delineating the moderating roles of FC and EHL, offering actionable insights for digital-age public health strategies.

## Introduction

### Background

Adolescent sugar intake has become a pressing public health issue in China, where teens increasingly consume sugar-sweetened beverages, milk-tea drinks, and confectionery readily available around schools and in urban retail settings [1]. Against dietary guidance that free sugars should provide less than 10% of total energy (with further benefits at <5%), intakes in many youth populations exceed these thresholds. Policy experiences abroad (eg, sugar-sweetened beverage taxes and reformulation in Mexico, the United Kingdom, and Chile) show that exposure is modifiable. Critically, high sugar intakes displace protective nutrients—fiber, calcium, iron, and high-quality protein—from whole grains, fruits, vegetables, and dairy, reinforcing a high-sugar, low-nutrient-density pattern. Globally, elevated sugar intake in youth is linked to overweight and obesity, and to early type 2 diabetes risk, nonalcoholic fatty liver disease, dental caries, and adverse cardiometabolic profiles. Framing China’s youth diets within this evidence underscores an urgent priority [2,3]: reduce free-sugar exposure while improving intake of key nutrients to prevent metabolic disease trajectories that begin in adolescence.

From a demographic and individual perspective, reducing free-sugar exposure is crucial for youth health. Authorities and public health agencies should deploy coordinated, standard-based interventions—taxing sugary beverages with consumption taxes graded by sugar density, restricting school food and public procurement standards covering beverage lists, sugar content thresholds, portion sizes, and retail locations; and routinely monitoring the progress of ingestion, sale, and recycling to limit adolescents’ exposure to high sugar [4]. However, without complementary behavioral changes, systemic-level measures are insufficient. At the individual level, adolescents should also establish glucose reduction awareness, such as replacing sugary drinks and ultra-processed snacks with water, slightly sweetened dairy products, whole grains, fruits, and vegetables, and maintaining regular dietary patterns that control controllable sugars. Because many adolescents are unable to establish glucose reduction behaviors fully, uncontrolled intake accelerates obesity and insulin resistance, leading to lifelong risks of type 2 diabetes, cardiovascular disease, nonalcoholic fatty liver disease, and tooth decay [5]. Therefore, health education that establishes glucose-control knowledge, motivation, and a sense of self-efficacy should be considered a core pillar of youth nutrition policies and practices.

Sugar-cutting trends, cultural influences, and patterns must be considered to understand collective behavior. Social media is key in promoting healthy behaviors, especially regarding reducing sugar [6,7]. Platforms such as WeChat (Tencent Inc), Weibo (Weibo Corporation), and TikTok (ByteDance Ltd) have a massive reach because there were 847 million Chinese internet users in June 2019, and the rise of these netizens marks the rise of highly internet- and information-empowered health information campaigns [8]. While international studies have explored the effects of social media on health behaviors, including glucose reduction, there remains a gap in understanding how glucose reduction works through social media in Eastern cultural settings. For example, the meta-analysis by Laranjo et al [9] suggests that the impact of social network interventions on health behaviors often lacks a strong theoretical basis and rigorous methodology. Maher et al [10] highlight that many social media–based interventions fail to incorporate robust theoretical frameworks, limiting their effectiveness. Although social media offers a promising platform for reducing sugar intake in China, research on its impact on eating habits remains limited. Therefore, further empirical research is needed to design a research framework with cultural relevance and a theoretical basis to effectively explore how social media plays a role in reducing sugar in China’s cultural environment.

Face concern (FC; mianzi) is a defining concept in Chinese culture, serving as a form of social currency deeply embedded in relational networks. It motivates alignment with valued behaviors and discourages actions that could invite social disapproval. In the context of adolescent health, face functions as an incentive for behaviors such as sugar reduction: adopting sustainable and controllable sugar reduction behaviors (SRBs) signals a sense of responsibility to peer groups and families [11]. Situated within the Chinese cultural context, this study integrates FC (mianzi) into the examination of how social media influences adolescent SRBs. This highlights a critical gap: existing models rarely specify FC as a mechanism linking digital stimuli to health behaviors. Therefore, we extend the stimulus-organism-response (SOR) framework by incorporating FC as a moderator in the pathway from social media exposure (stimulus) to conformity and health consciousness (HC; organismic state), ultimately influencing SRB (response). Empirically, our analysis provides evidence from Chinese youth, supporting the practical value of social media–based health communication in this context and broadens the cultural applicability of the SOR model. More broadly, the findings underscore that effective health promotion hinges on aligning technological affordances with deeply rooted social values, thereby enhancing both theoretical precision and real-world relevance.

### Theoretical Framework and Research Hypothesis

#### Theoretical Framework

The SOR model, proposed by Mehrabian and Russell [12], offers a comprehensive framework for understanding how individuals respond to environmental stimuli. It consists of 3 components: stimulus (external cues or information), organism (individual attributes such as psychological, cognitive, and emotional factors), and response (resulting behavioral, cognitive, or emotional changes). Widely used in fields such as consumer behavior, health promotion, and digital interventions, the SOR model is particularly effective for studying phenomena driven by rapidly emerging external cues, such as social media exposure [13-16]. Unlike models such as the health belief model and theory of planned behavior, which focus on belief-based assessments (eg, perceived risks and attitudes), the SOR framework highlights the interplay of both cognitive and emotional responses in shaping behavior [17,18]. In addition, while social cognitive theory incorporates self-efficacy and observational learning, it lacks a formal pathway from stimulus to response [19]. Similarly, the Capability, Opportunity, Motivation, and Behaviour Model provides a functional system-level perspective but overlooks the microprocesses of exposure and arousal inherent to digital environments [20]. This study extends the SOR model by integrating social media sugar-reduction cues as the stimulus, HC and conformity as distinct organism states, and SRB as the response, thus enhancing the model’s theoretical precision and relevance to culturally specific health behaviors in digital contexts.

This study integrates FC (mianzi) into the SOR framework to explain, within an East Asian cultural context, how digital media shape Chinese adolescents’ SRBs. Whereas previous work typically applies SOR to generic media effects, our integration specifies a culturally grounded “influence effector” on the S→O pathway and analytically separates 2 organismic states—conformity (seeking social approval) and HC (an intrinsic valuation of health). By delineating how social media stimuli are translated into cognitive and affective organismic responses under the amplifying or attenuating force of FC, the model decenters individualist assumptions that dominate behavior-change theory, builds a theoretical bridge between cultural psychology and digital health communication, and offers an actionable, theory-driven pathway for culture-informed health behavior practice. In summary, coupling SOR with a core Chinese cultural norm—face—yields a novel, generalizable, and adaptable framework for modeling how culture and media jointly convert online cues into offline health action. Combining SOR with facial attention also provides an actionable, realistic framework for public health that can be applied in other collectivist or honor-code settings outside of China [21]; it is also compatible with policy tools (school standards and platform guidelines) and regular assessments. By combining cultural specificity with digital scalability and rigorous causal logic, the model advances the classical “SOR” theory and informs interventions that, in communication, are more likely to be successful than those not. Public health and behavioral science fields have the dual contribution of “significance-reality” [22]. The face-based extended “SOR” theoretical model has cross-cultural implications for conceptual innovation and for designing, positioning, and evaluating the practical utility of social media–based health promotion.

#### Research Question and Hypothesis

Young individuals generally lack disease knowledge and experience, which may stem from their limited exposure to illnesses. This gap affects their comprehension and application of health information [23]. Moreover, the lack of disease experience can lead to difficulties in evaluating and selecting health information [24], insufficient perception of disease threat, and an inability to form clear health attitudes and beliefs [25]. In this context, when confronted with health-related issues or decisions, young people may conform to the views and choices propagated by various social media users. This tendency is particularly pronounced given the proliferation of health information on Chinese social media platforms in recent years [26], the widespread application of social media in health care, and a strong collective culture that emphasizes group goals and interpersonal harmony, leading to increased conformity [27]. Therefore, conformity among Chinese youth on social media offers a compelling explanation for the sugar reduction trend. Beyond short-term conformity, sustained exposure to health information may also gradually enhance users’ HC, further influencing their behaviors [28]. Thus, conformity in the social media environment and the rise in HC represent 2 interrelated pathways. Accordingly, the research questions are as follows:

1. RQ1: Does the trend of sugar reduction among Chinese youth stem from conformist decision-making under the influence of social media, or is it an outcome of enhanced HC nurtured by prolonged exposure to the social media? Should there be an interplay between the 2, which factor takes the lead?
2. RQ2: Does the SRB of Chinese youth exhibit any distinctive influences and mechanisms specific to the Eastern cultural backdrop?

Health outlook among China’s youth is closely tied to social media—a powerful driver of change that distinguishes their attitudes from those of older cohorts [29]. In the context of digital natives, social media has become the primary arena where Chinese youth acquire, exchange, and reproduce health information [30]. On these platforms, an endless stream of rapidly updated sugar-reduction content—ranging from influencers’ low-sugar recipes and peers’ daily check-in posts to algorithm-driven short videos—collectively functions as a continuous behavioral cue [31]. From the perspective of the theory of planned behavior [32], these external cues enhance attitudes, subjective norms, and perceived behavioral control, ultimately increasing the likelihood of SRB. Moreover, the concurrent rise of the “sugar control” agenda in both policy and commercial discourses in China in recent years has endowed sugar-reduction discussions on social media with both informational and normative significance, further amplifying their influence on young people’s dietary decisions [33,34]. Therefore, the more frequently young people use social media and the more deeply they are engaged, the more likely they are to translate the sugar-reduction information they receive online into actual SRB offline. Accordingly, Hypothesis H1 is formulated as: social media usage (SMU) positively influences SRB.

Social media has become the primary information environment in which individuals make conformity-based decisions [35,36]. This manifests as informational conformity [37]: when uncertain or unfamiliar, users turn to others’ opinions to shape their own views and behaviors. For example, given their limited experience with illness and insufficient ability to evaluate health information, Chinese youth are more likely to rely on cues from peers on social media as a shortcut for decision-making [38]. On the other hand, young people are more susceptible to the influence of a sense of belonging and identity [39], which leads to normative conformity effects. For instance, the massive user-generated content on platforms—such as sugar-reduction check-ins, demonstrations by bloggers, and rankings—collectively create a descriptive norm of “everyone is reducing sugar” and an injunctive norm that “reducing sugar is healthy” [40]. Based on the emphasis of interpersonal harmony in collectivist culture, FC further amplifies this normative pressure: the deeper the social media embedding, the more individuals care about approval within their social circles, and the stronger the conformity intention [9]. The high intention to conform prompts young people to reduce their intake of sugary drinks and choose low-sugar foods to meet group expectations and gain online recognition. Thus, the intention to conform transforms short-term online following into offline sugar reduction actions. Therefore, we propose Hypothesis H2: conformity mediates the positive effect of SMU on SRB.

Nowadays, social media is not only the primary channel for young people to obtain health information, but also provides a continuous stream of popular science short videos, influencer recipes, health app check-in records, and peer experience sharing, all of which together create an “informational immersion” environment [41]. In this environment, users are passively or actively exposed to a large amount of content about the dangers of excessive sugar intake, the benefits of a low-sugar diet, and self-monitoring skills. This exposure continuously awakens their attention to their own health status and enhances their health self-awareness, health vigilance, and health engagement [42]. Previous research has confirmed that the higher the frequency of social media use, the higher the level of HC among individuals [43]. Moreover, the enhancement of HC significantly promotes a variety of healthy behaviors, including sugar reduction [44]. Therefore, SMU indirectly drives SRB among Chinese youth by enhancing HC, forming a chain of “usage-consciousness-behavior.” Based on this mechanism, we propose Hypothesis H3: HC mediates the positive effect of social media use on SRB among Chinese youth.

In the context of China’s collectivist culture intertwined with “FC,” individuals’ behaviors on social media are not only about personal preferences but also about online identity and group recognition. As the intensity of use deepens, users become more embedded in online social circles, and their FC is activated: they increasingly care about evaluations, likes, and shares within their social circles, worrying that deviating from group norms will “lose face” [45]. Empirical studies have indicated that the higher the level of FC, the more inclined individuals are to conform to popular topics to maintain their online image [46]. Therefore, FC plays a positive moderating role between social media use and conformity intention: when the level of FC is high, the positive effect of social media use on conformity is significantly amplified, thereby intensifying the trend-following conformity behavior of sugar reduction. Thus, we put forward Hypothesis H4: FC plays a moderating role between SMU and conformity.

eHealth literacy (EHL) has a significant impact on individuals’ health behaviors [47]. The translation of HC into actual SRB hinges on the youth’s capacity to process and apply social media health information [48]. Individuals possessing higher EHL can effectively locate, critically evaluate, and implement evidence-based guidance on low-sugar diets, thereby converting risk awareness into concrete sugar-reduction actions [49]. In contrast, those with low EHL, though aware of health risks, may struggle to act due to difficulties in identifying reliable information or limited implementation skills. Consequently, EHL positively moderates the relationship between HC and SRB: higher levels strengthen this association, whereas lower levels weaken it. We therefore propose Hypothesis H5: EHL plays a moderating role between HC and SRB.

**Figure 1.**
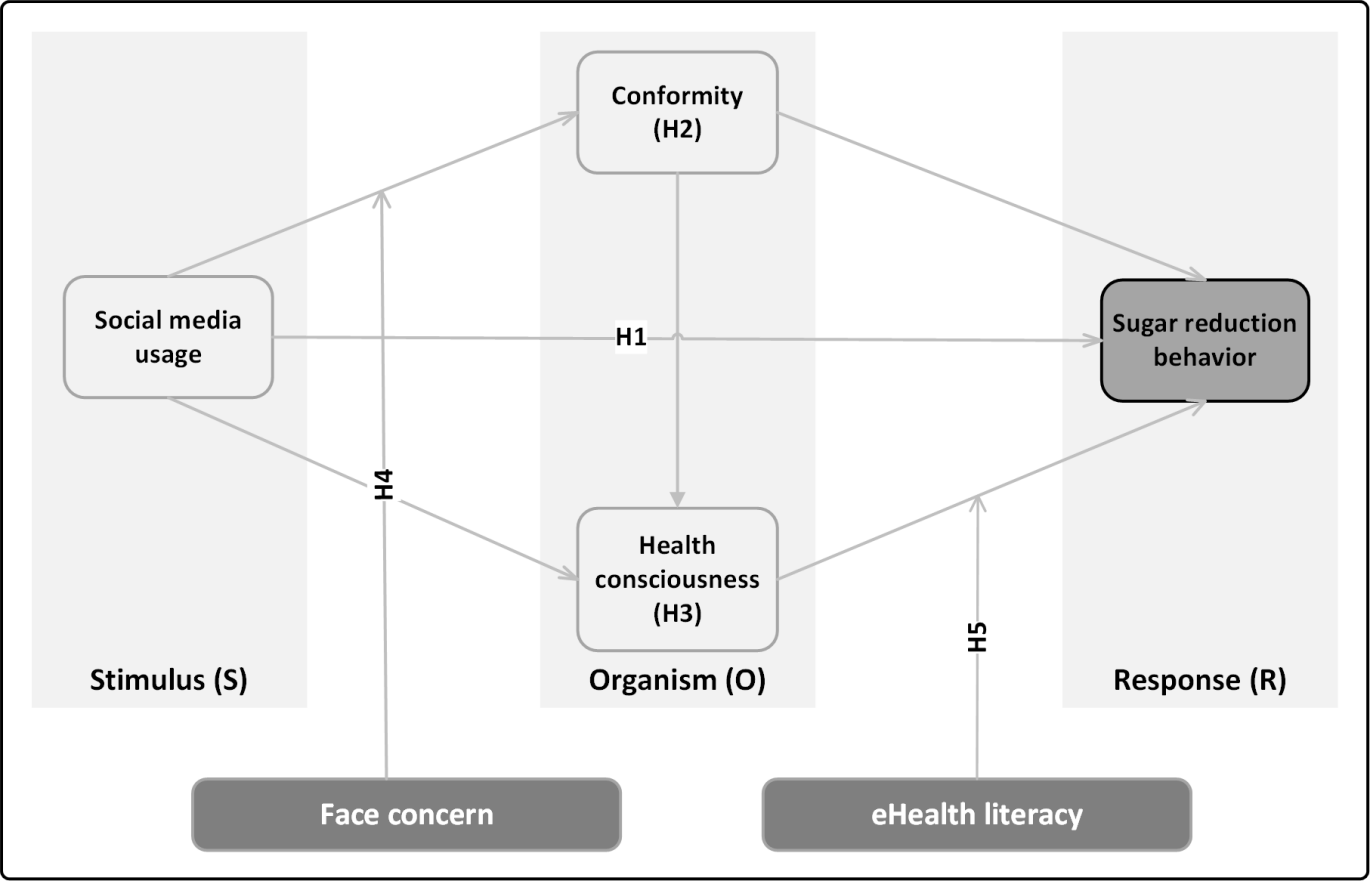
Conceptual model and research hypotheses.

Drawing on the aforementioned analysis, as provided in Figure 1, H1 examines the direct effect of SMU on SRB. H2 and H3 examine the parallel mediating roles of conformity and HC, respectively. H4 and H5 test the moderating roles of FC and EHL, respectively. In addition, we investigate the serial mediating path linking SMU, conformity, HC, and SRB; this portion is exploratory, and no directional hypothesis is proposed.

## Methods

### Design and Recruitment

Our study focused on Chinese youth. The World Health Organization (WHO) categorizes youth as ages 15‐44 years [50], the European Union as 18‐35 years [51], and the National Bureau of Statistics of China as 15‐34 years [52]. This study defines youth as individuals aged 15‐35 years, with questionnaire filters matching this range. Participants were recruited online via Questionnaire Star (Changsha Ranxing Information Technology Co Ltd), a survey company whose panel comprises 2.6 million registered panelists across China. We developed a structured questionnaire for this study (Multimedia Appendix 1). We applied integrated safeguards to reduce selection bias to an acceptable level. First, the platform’s random-allocation function ensured that all eligible respondents had an equal opportunity to participate, and eligibility screening questions excluded 3.8% of invalid responses. Second, IP and device verification removed 1.9% of VPN-based cross-regional access and 2.3% of duplicate submissions. Third, we computed post-stratification weights for age, sex, education, and region and re-estimated the structural equation model (SEM); path coefficients changed by <0.02, and significance levels remained intact, indicating that the substantive conclusions are robust to remaining demographic imbalances. The weight values and poststratification-weighted SEM results are provided in Multimedia Appendix 2.

To mitigate social desirability and self-report bias, we implemented a series of systematic controls. First, we embedded reverse-scored items and logic consistency checks in the questionnaire; responses failing these checks (3.8% of cases) were excluded. The survey emphasized behavioral measures rather than direct attitude items. Calibration statements (eg, “Carbohydrates are one of the essential nutrients for the human body”) and honesty test items (eg, “Sugar is a tasteless substance”) were used to detect inattentive or insincere respondents.

Furthermore, to meet analytical requirements, the required sample size was estimated using G*Power (version 3.1.9.7; Heinrich-Heine-Universität Düsseldorf) based on Cohen [53] effect size conventions, with the SRB score as the outcome variable. For the 5-group (Education) ANOVA, we specified Cohen *f*=0.25, with power=0.95 and *α*=.05, yielding a minimum sample size of 305. For the independent *t* test (liberal arts vs science and engineering), we maintained these parameters but used Cohen *d*=0.25 (equivalent to *f*=0.25 in standardized terms), which required a minimum sample size of 834. The discrepancy in sample sizes (305 vs 834) arises because ANOVA’s global test efficiency reduces requirements for multigroup comparisons, whereas *t* tests require larger samples to control family-wise error rates during multiple pairwise comparisons (adjusted α=.01 via Bonferroni correction). Reconciling both criteria, we conservatively adopted the *t* test–derived (n=883), ensuring robustness against type II errors even if true effect sizes decrease to *f*=0.20 (ANOVA minimum, n=394) or d=0.20 (*t* test minimum, n=650). The initial data collection phase took place on June 20, 2024, with a preliminary survey yielding 172 responses from the Questionnaire Star platform, of which 144 were deemed valid. After assessing the questionnaire’s reliability and validity, we implemented necessary adjustments. The formal second phase of the survey was launched between July 8 and August 8, 2024, resulting in 1053 collected questionnaires. Excluding invalid responses based on calibration scale, honesty check, logical consistency check, and other quality control measures, we retained 883 valid questionnaires, achieving a valid response rate of 83.9% (883/1053), which satisfies the minimum sample size criteria based on the *t* test–derived minimum requirement of 834.

The demographic characteristics of the sample are provided in Table 1. It is noticeable that participants were predominantly aged 18 to 30 years (803/883, 90%), with a slight female preponderance (460/883, 52.1%) over males (423/883, 47.9%). The majority (688/883, 78%) had a bachelor’s degree or higher. An impressive 849 (96%) participants had been engaged with social media for more than 3 years. The geographical spread showed a higher urban (567/883, 64.2%) than rural (316/883, 35.8%) representation. Regarding BMI, which is tied to sugar intake, the majority (654/883, 74.1%) were of normal weight, and a small fraction (74/883, 8.4%) were classified as overweight. In summary, the sample’s demographic profile fits the study’s demographic focus.

**Table 1.** Sociodemographic characteristics of the research sample (N=883).

Characteristic	Value, n (%)
Sex	
Male	423 (47.9)
Female	460 (52.1)
Education	
Below junior high school level	7 (0.8)
High school or secondary vocational education	108 (12.2)
Junior college	80 (9.1)
Undergraduate	602 (68.2)
Postgraduate and above	86 (9.7)
Age (years)	
15‐18	9 (1.0)
18‐24	577 (65.3)
24‐30	226 (25.6)
30‐35	71 (8)
Professional background	
Liberal arts	400 (45.3)
Science and engineering	483 (54.7)
BMI	
Underweight (BMI <18.5)	155 (17.5)
Normal weight (18.5≤ BMI <24.9)	654 (74.1)
Overweight (BMI ≥25)	74 (8.4)
Social media usage duration	
≤3 years	34 (3.9)
3‐5 years	274 (31.0)
5‐10 years	411 (46.5)
>10 years	164 (18.6)
Family residence	
Countryside	316 (35.8)
Cities and towns	567 (64.2)

### Measurement

The study used a 5-point Likert scale for all measurements, adapted from validated scales in recent academic literature. Following Brislin’s protocol [54], 2 bilingual translators independently produced Chinese versions, reconciled discrepancies, and a third translator back-translated blindly; iterations resolved semantic mismatches. A pilot study with 144 demographically representative Chinese youth participants refined scale item wording to ensure linguistic equivalence and cultural appropriateness.

#### SMU

The SMU scale, consisting of 6 items, adapted from the Social Networking Site Use Scale developed by Ellison et al [55], originally developed for Facebook (Meta Platforms Inc), was adapted for the Chinese sample. Ellison et al [55] first 2 items—friend count and daily usage time—were specifically designed for Facebook and were poorly aligned with China’s WeChat-centric ecosystem; these items were excluded, and the remaining 6 were culturally adapted. The original scale’s context was adapted from Facebook to Chinese social media platforms, with items such as “Using social media is a part of my life” and “I spend most of my time on social media.” This scale demonstrated acceptable internal consistency (Cronbach *α*=0.79), with a mean score of 3.61 (SD 0.67).

##### SRB

To focus the measurements and research on low-carbohydrate diet practices while using established lifestyle frameworks, we adapted 3 items in the field of nutrition of health-promoting lifestyle profiles II (Walker et al [56]; Chinese version: Huang and Chiu [57]) to capture hypoglycemic behaviors (SRB)—covering product choices and the effects of SRB on health-promoting lifestyle profiles, actively reduce and label use (eg, replace “I choose low-fat, low-saturated-fat, low-cholesterol foods” with “I choose low-sugar foods and beverages” and adding items such as “I deliberately reduce high-sugar diets and sugary foods” and “I pay particular attention to sugar content labels on food and beverage packages”). To ensure structural validity and situational appropriateness, we implemented a phased adaptation-validation protocol. First, a multidisciplinary expert assessment (nutrition, adolescent health, health communication, and psychometrics) was conducted to demonstrate that the scale content validity indicators were in line with the routine, while also conforming to the appropriateness of the Chinese cultural context. Second, cognitive interviews were conducted with adolescents to assess comprehension and reaction processes, followed by minor wording improvements. Finally, the validation of internal consistency, convergent validity, and differential validity was performed, and all indicators reached a priori thresholds, supporting the adaptive item as a specific measurement item of this questionnaire. The scale demonstrated good reliability (Cronbach *α*=0.85), with a mean of 3.62 (SD 0.99; Table B1 in Multimedia Appendix 3).

##### Conformity

To measure conformity in adolescents’ SRBs, we adapted the Informational and Normative Conformity Scale (SKI-N) by Opozda-Suder et al [58], shifting its focus from offline peer relationships to social media interactions. The original 2-factor structure was retained, and items were reworded for the digital context. For example, “I believe it is important to align with the majority on social media, which often leads me to adopt the majority’s viewpoint” (normative) and “When I am unsure of my own views, I tend to refer to others’ opinions on social media” (informational), reflecting digital influences such as platform cues and peer-driven content. To ensure construct validity, we followed a rigorous validation process. First, expert assessments from adolescent health, cultural psychology, and psychometrics were conducted to evaluate content relevance and cultural appropriateness. Second, the scale was translated and back-translated, followed by pilot testing with adolescents to refine clarity and ensure cultural alignment. Finally, psychometric tests (exploratory factor analysis and confirmatory factor analysis) were performed to confirm the 2-factor structure, internal consistency, and convergent-discriminant validity. The adapted SKI-N demonstrated strong validity, reliability, and cultural applicability for measuring conformity in social media environments (Cronbach *α*=0.92; mean 3.47, SD 0.88).

### HC

The measurement of HC was based on the Health Consciousness Scale (HCS) developed by Gould [35], which comprises 4 dimensions and 9 items. The dimensions include health self-consciousness with items such as “I reflect about my health a lot;” health involvement with items such as “I’m very involved with my health;” health self-monitoring with items such as “I’m aware of the state of my health as I go through the day;” and health alertness, with items such as “I’m alert to changes in my health.” The scale demonstrated strong reliability (Cronbach *α*=0.90) and acceptable overall scores (mean 3.95, SD 0.61).

### EHL

EHL was assessed using the EHL Scale developed by Norman and Skinner [48], which measures individuals’ perceived skills in finding, evaluating, and applying online health information. Example items include “I know how to find helpful health resources online” and “I know how to use the internet to answer my health questions.” Responses were recorded on a 5-point Likert scale (1=strongly disagree to 5=strongly agree), with higher scores indicating greater EHL. The scale demonstrated strong reliability (Cronbach *α*=0.90) and an acceptable overall mean score of 3.95 (SD 0.60).

### FC

FC was measured using a scale adapted from Chan et al [59], which was originally derived from Cocroft and Ting-Toomey [60] and White et al [61]. The scale assesses individuals’ sensitivity to maintaining face in social interactions. Example items include “I care about others’ attitudes toward me” and “I am very pleased to receive respect.” The scale demonstrated good internal consistency in this study (Cronbach α=0.823; mean 3.90, SD 0.65).

### Data Analysis Methods

As the multivariate normality test using Stata (version 15.0; StataCorp LLC) indicated that the study’s sample does not adhere to a multivariate normal distribution (Mardia mSkewness=9.57; *P*<.001 and Mardia mKurtosis=95.55; *P*<.001), partial least squares–based SEM was adopted, as it exhibits robustness with highly skewed data. Furthermore, as suggested by Hair et al [62], partial least squares structural equation modeling is well-suited for complex models with multiple constructs and relationships, allowing for both explanation and prediction. SmartPLS (version 4.0; SmartPLS GmbH) was used for model construction, while SPSS (IBM Corp) supported descriptive statistics, *t* tests, reliability analysis, ANOVA, Pearson correlation, and exploratory factor analysis. To address inflated type I error risks from multiple comparisons (eg, age group ANOVA with 4 pairwise tests), Bonferroni correction was applied by adjusting α to .0125 (.05/4) for post hoc analyses. For *t* tests involving multiple dependent variables (eg, SRB), false discovery rate control was implemented using the Benjamini-Hochberg procedure with Q=0.05. SmartPLS (version 4.0) is also used to evaluate the measurement’s reliability and validity, as well as to assess and determine the model’s fitness. Evaluation indicators include path coefficients (β), R-squared (*R*²), standardized root-mean-square residual, chi-square, normed fit index, predicted R-squared (Q²predict), root-mean-square error, mean absolute error, Akaike Information Criterion, Unbiased Akaike Information Criterion, final prediction error, Bayesian Information Criterion, Geweke-Meese criterion, and Hannan-Quinn criterion. For the moderation effect analysis, we included 2 interaction items in the structural equation, SMU × FC and HC × EHL. Simple slope graphs were plotted based on the significance and magnitude of the interaction term coefficients.

### Ethical Considerations

The study protocol was reviewed and approved by the Ethics Committee of Chang Gung University (approval code: IRB no. 20240121B0; approval date: January 21, 2024). All procedures complied with the ethical standards of the institutional and national research committees and adhered to the principles of the Declaration of Helsinki. All participants were fully informed of the study purpose, procedures, potential risks, and their right to withdraw at any time without penalty. Written informed consent was obtained before data collection.

To protect participants’ privacy and confidentiality, no identifying information (eg, names, student IDs, contact details, and IP addresses) was stored. All responses were anonymized at the point of data entry, and data were used solely for research purposes. Only the research team had access to the deidentified dataset, which was securely stored on password-protected servers. Participants did not receive any monetary or nonmonetary compensation for their participation.

## Results

### Differential Test of Demographic Characteristics

Using SPSS (version 25.0) software, the study conducted *t* tests and ANOVA to assess demographic variations in SRBs within the research sample. We observed significant disparities in SRBs across sex (*P*=.05), age (*P*<.001), and professional background (*P*<.001). Differential analyses revealed significant demographic variations in SRB. Age exerted a substantial effect (*F*_3,879_=6.28; *P*<.001), with Bonferroni-adjusted pairwise comparisons confirming lower SRB among 15‐ to 18-year-olds (mean 3.51, SD 1.05) versus 24‐ to 30-year-olds (mean 3.81, SD 0.80; adj *P*=.004). Education levels did not differ significantly after correction (all adjusted *P*>.09), although marginal uncorrected trends emerged between below junior high (mean 3.24, SD 0.37) and junior college (mean 3.89, SD 0.92; *P*=.02). BMI comparisons revealed no significant group differences (all adj *P*>.126) despite nominal trends toward higher SRB in overweight (mean 3.83, SD 1.01) versus normal-weight participants (mean 3.61, SD 0.97; *P*=.02). Males reported significantly higher SRB scores than females (male: mean 3.67, SD 0.93 vs female: mean 3.56, SD 1.04; *t*_881_=2.00; *P*=.05), science and engineering majors outperformed liberal arts counterparts (science and engineering: mean 3.84, SD 0.82 vs liberal arts: mean 3.45, SD 1.08; *t*_881_=6.13; *P*<.001). Residential location comparisons showed null effects (*P*=.78). Complete statistical outcomes are provided in Table 2.

**Table 2.** Differential testing of demographic characteristics in sugar reduction behavior (N=883).

Characteristic	Value, mean (SD)	Statistic or pairwise	*P* value	Adj *P* value
Sex		*t*_881_=2	.05	—^a^
Male	3.67 (0.93)			
Female	3.56 (1.04)			
Education				
Below junior (BJ) high school	3.24 (0.37)	*F*_4878_=2.11	.09	—
Vs HS	—	—	.03	.16
Vs JC	—	—	.02	.09
Vs UG	—	—	.05	.23
Vs PG	—	—	.02	.12
High school (HS)/secondary vocational education	3.66 (0.74)	—	—	—
Vs JC	—	—	.57	1.00
Vs UG	—	—	.32	1.00
Vs PG	—	—	.46	1.00
Junior college (JC)	3.89 (0.92)	—	—	—
Vs UG	—	—	.19	.95
Vs PG	—	—	.28	1.00
Undergraduate (UG)	3.58 (1.04)	—	—	—
Vs PG	—	—	.09	.45
Postgraduate (PG) and above	3.62 (1.01)	—	—	—
Age (years)		*F*_3879_=6.28	<.001	—
15‐18	3.51 (1.05)	—	—	—
18‐24	3.70 (0.94)		—	—
Vs 15-18	—	—	.002	.008
24‐30	3.81 (0.80)	—	—	—
Vs 15-18	—	—	<.001	.004
30‐35	3.95 (0.81)	—	—	—
Vs 15-18	—	—	<.001	.004
BMI		*F*_2880_=2.27	.10	—
Underweight (BMI <18.5)	3.53 (1.03)	—	—	—
Vs normal weight	—	—	.40	1.00
Vs overweight	—	—	.08	.47
Normal weight (18.5≤ BMI <24.9)	3.61(0.97)	Versus overweight	.02	.13
Overweight (BMI ≥25)	3.83 (1.01)	—	—	—
Professional background		*t*_881_=−6.13	<.001	—
Liberal arts	3.45 (1.08)			
Science and engineering	3.84 (0.82)			
Family residence		*t*_881_=0.31	.78	—
Countryside	3.64 (0.94)			
Cities and towns	3.62(1.02)			

a Not required.

### Validity and Common Method Bias Test

The measurement model demonstrated satisfactory convergent validity (all average variances extracted >0.50) and discriminant validity according to the Fornell-Larcker criterion [63]. Common method bias remained within acceptable limits (Harman single factor=22.98%; all variance inflation factors <3.3, per Kock [64]). Detailed validity metrics and bias test results are provided in Tables B2 and B3 in Multimedia Appendix 3.

### Model Fit

The hypothesized model (Figure 1) demonstrated superior predictive accuracy over alternative configurations, achieving optimal performance balance with lower prediction errors (root-mean-square error=0.944 and mean absolute error=0.735), higher predictive relevance (Q²=0.113), and significantly better outcomes in cross-validated testing (loss=0.945; Δ loss=−0.002 vs sequential mediation; *P*=.32). This configuration satisfied critical fit thresholds (standardized root-mean-square residual=0.072<0.08 benchmark), confirming the theoretical and empirical adequacy of our extended SOR framework for explaining SRBs, with comprehensive model fit indices and cross-validated testing results provided in Tables C1 and C2 in Multimedia Appendix 3.

### Hypothesis Testing

Hypothesis testing was conducted using SmartPLS (version 4.0) to construct an SEM in a 2-step process. Initially, a chained mediation model without moderating paths was estimated. Subsequently, the model was extended to include moderating effects. Control variables included sex, major, education level, BMI, and self-perceived health status. The self-perceived health status item is phrased as “How do you rate your health condition in the past six months?” with response options: very good, good, average, poor, and very poor.

### Direct Effect

A bootstrapping procedure, with 5000 resamples, was used to estimate the path coefficients and significance levels of the relationships between variables, as provided in Table 3 and Table C3 in Multimedia Appendix 3. The *R*^2^ of the model’s dependent variable SRB is 0.376, which aligns with the criteria established by Chin [65] and signifies a moderate degree of explanatory power.

**Table 3.** Direct effect path coefficients.

Path	β (95% CI)	SE^a^	t^b^	*P* value	Findings
Social media usage→conformity	0.51^c^ (0.45 to 0.57)	0.032	15.86	<.001	—^d^
Social media usage→sugar reduction behavior	0.08 (0.01 to 0.16)	0.039	2.11	.04	H1 supported
Conformity→sugar reduction behavior	0.14 (0.07 to 0.21)	0.037	3.78	<.001	—
Social media usage→health consciousness	0.35 (0.28 to 0.43)	0.037	9.58	<.001	—
Health Consciousness→sugar reduction behavior	0.50 (0.44 to 0.56)	0.030	16.37	<.001	—
Conformity→health consciousness	0.05 (–0.03 to 0.13)	0.042	1.30	.19	Parallel mediation

a In the partial least squares structural equation modeling (PLS-SEM) model conclusion report, SE denotes the standard error of coefficients calculated using the bootstrap method, and this applies consistently throughout.

b In PLS-SEM, degrees of freedom for *t* values are not reported, as statistical inference relies on bootstrapping, which directly provides *t* and *P* values for each path coefficient. This approach eliminates the need for conventional degrees of freedom, and we apply the same procedure in later analyses.

c Considering the accuracy of PLS-SEM results reporting, retain 3 decimal places, and the same applies subsequently.

d Not applicable.

As provided in Table 3, the direct effect of SMU on SRB is positive (β=.08, 95% CI 0.01-0.16; *P*=.04), supporting H1. SMU exerts a significant positive influence on conformity (β=.51, 95% CI 0.45-0.57; *P*<.001). Conformity significantly and positively predicts SRB (β=.14, 95% CI 0.07-0.21; *P*<.001). In addition, SMU positively affects HC (β=.35, 95% CI 0.28-0.43; *P*<.001). HC also positively predicts SRB (β=.50, 95% CI 0.44-0.56; *P*<.001).

**Figure 2.**
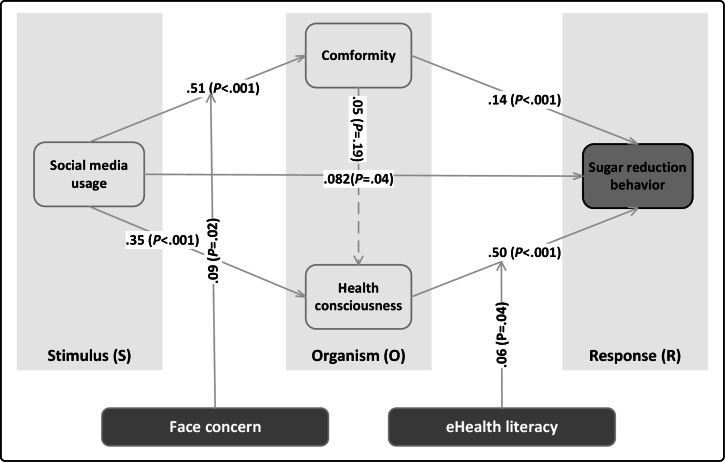
Result of the structural model.

Referring to Figure 2, the path coefficient for the relationship between conformity and HC is positive but not significant (β=.05, 95% CI −0.03 to 0.13; *P*=.19). Among the control variables, professional background, BMI, and self-perceived health status significantly predict preventive behaviors, whereas sex and education level do not. Specifically, individuals with a science and engineering background and those with higher BMI are more likely to engage in SRBs. In addition, individuals with lower self-perceived health status are more inclined to undertake SRBs.

### Dual Mediation Path of SRBs

Mediation effects were examined using the bootstrap method, with the results provided in Table 4 [66].

In the mediation pathway SMU→conformity→SRB, the total effect of SMU on SRB is 0.34 (95% CI 0.28-0.40; *P*<.001). The specific indirect effect is significant at 0.07 (95% CI 0.03 to 0.10; *P*<.001), with the mediation effect accounting for a Variance Accounted For of 21.05% This suggests that conformity partially mediates the relationship between SMU and SRB, constituting partial mediation, and supports Hypothesis H2.

In the mediation pathway SMU→HC→SRB, the total effect of SMU on SRB is 0.38 (95% CI 0.32-0.44; *P*<.001). The specific indirect effect is significant at 0.18 (95% CI 0.14-0.22; *P*<.001), with the mediation effect accounting for a Variance Accounted For of 51.5%. This indicates that HC mediates the relationship between SMU and SRB, establishing partial mediation and supporting Hypothesis H3.

The specific indirect effect in the mediation pathway SMU→conformity→HC→SRB is not significant (β=.01, 95% CI −0.01 to 0.04; *P*=.19). The direct effect SMU→SRB is significant at the 5% level (β=.08, 95% CI 0.01-0.16; *P*=.04). The exploratory serial mediation effect was not supported.

**Table 4.** Mediation effects test.

Path	β (95% CI)^a^	SE	*P* value	VAF^b^	Findings	Mediation type
Total effect of social media usage→sugar reduction behavior	0.34 (0.28 to 0.40)	0.033	<.001	—	—	—
Total effect of social media usage→health consciousness	0.38 (0.32 to 0.44)	0.031	<.001	—	—	—
Total effect of conformity→sugar reduction behavior	0.17 (0.08 to 0.25)	0.044	<.001	—	—	—
Direct effect of social media usage→sugar reduction behavior	0.08 (0.01 to 0.16)	0.039	.04	—	—	—
Total indirect effect of social media usage→sugar reduction behavior	0.26 (0.21 to 0.31)	0.025	<.001	76%	—	—
Specific indirect effect						
social media usage→conformity→sugar reduction behavior	0.07 (0.03 to 0.11)	0.019	<.001	21.05%	H2 supported	Partial
social media usage→health consciousness→sugar reduction behavior	0.18 (0.14 to 0.22)	0.021	<.001	51.5%	H3 supported	Partial
social media usage→conformity→health consciousness	0.03 (–0.02 to 0.07)	0.021	.19	7.09%	—	—
Conformity→health consciousness→sugar reduction behavior	0.03 (–0.01 to 0.07)	0.021	.20	16.36%	—	—
social media usage→conformity→health consciousness→sugar reduction behavior	0.01 (–0.01 to 0.04)	0.011	.20	4.09%	—	—

a Due to space constraints, the results for the coefficients of control variables in the mediation and moderation effects are omitted, and the same applies to the following reports.

b Variance Accounted For (VAF) measures the proportion of variance explained by the mediator in the relationship between the independent and dependent variables. Ranging from 0 to 1, a higher VAF reflects a stronger mediation effect. According to Hair 66][, VAF>80% indicates full mediation, 20%<VAF<80% shows partial mediation, and VAF<20% indicates no mediation.

### Moderation Effect Verification

Pursuant to Hypotheses H4 and H5, moderation effect models were established for the moderated paths of SMU→conformity and HC→SRB by incorporating interaction terms between the moderating variables and the independent variables. Moderation was inferred when interaction terms reached significance.

**Figure 3.**
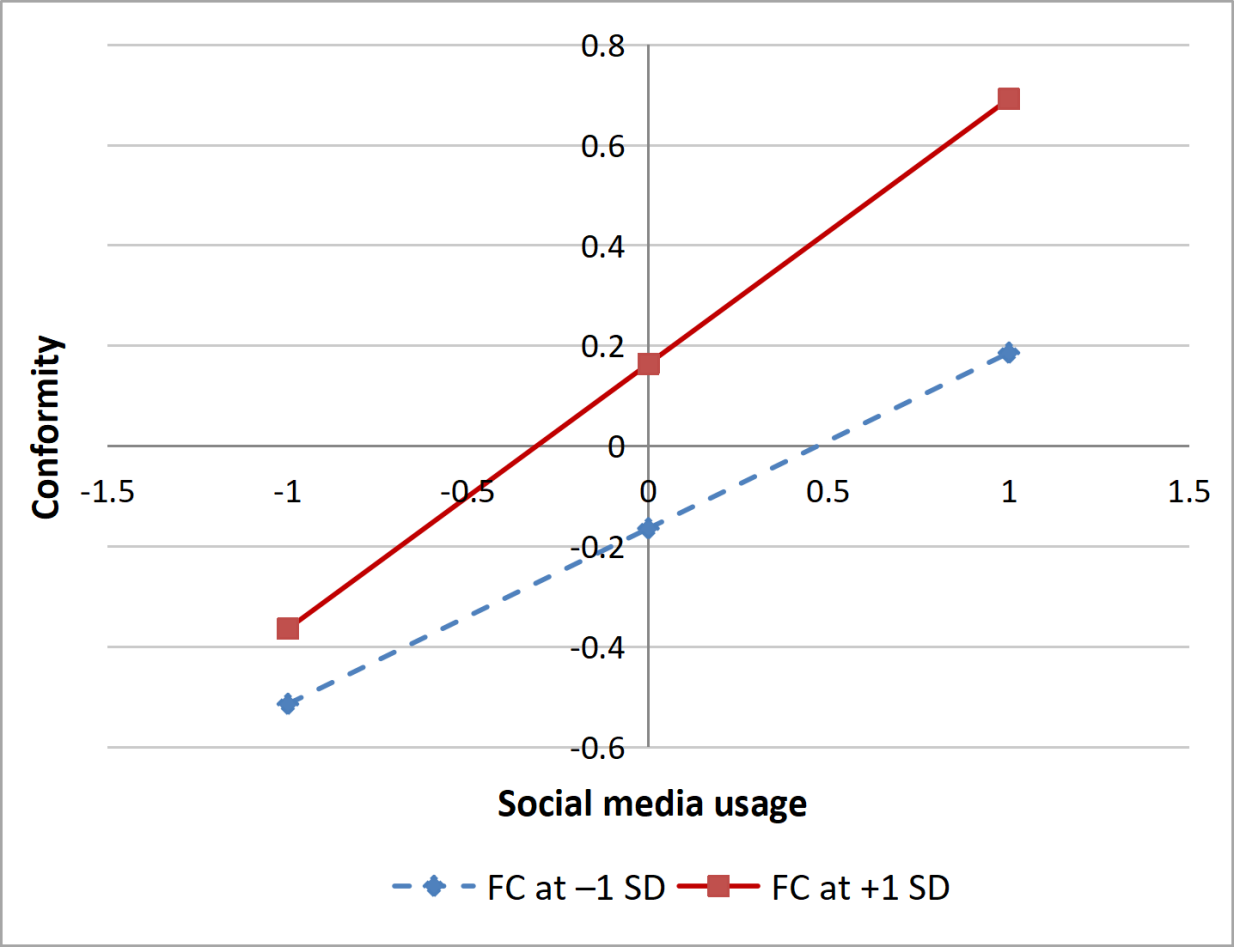
Face concern (FC) moderating effect diagram.

Table 5 shows that the interaction term between FC and SMU significantly and positively influences conformity (β=.09, 95% CI 0.01-0.17; *P*=.02), suggesting that FC positively moderates the impact of SMU on conformity, thereby validating Hypothesis H4. In addition, the interaction term between EHL and HC significantly and negatively affects SRB (β=.06, 95% CI 0.00-0.10; *P*=.04), indicating that EHL negatively moderates the process through which HC leads to SRB, thus confirming Hypothesis H5. Simple slope analyses were conducted using the mean of dispositional face concern plus or minus 1 SD, with the results provided in the following Figure 3.

**Table 5.** Moderation effects test.

Moderated path and relationship	β (95% CI)	SE	t	*P* value	Findings
FC^a^ moderates, SMU^b^→conformity					H4 supported
SMU^b^→conformity	0.44 (0.36 to 0.51)	0.038	11.62	<.001	
FC→conformity	0.16 (0.08 to 0.25)	0.041	3.98	<.001	
FC × SMU→conformity	0.09 (0.01 to 0.17)	0.039	2.28	.02	
FC at −1 SD	0.35 (0.23 to 0.46)	0.059	10.12	<.001	
FC at +1 SD	0.53 (0.43 to 0.62)	0.049	9.14	<.001	
EHL^c^ moderates, HC^d^→SRB^e^					H5 supported
HC→SRB	0.51 (0.42 to 0.59)	0.042	12.06	<.001	
EHL→SRB	−0.00 (−0.01 to 0.01)	0.047	0.02	.98	
EHL × HC→SRB	0.06 (0.00 to 0.01)	0.026	2.06	.04	
EHL at −1 SD	0.45 (0.36 to 0.55)	0.049	9.24	<.001	
EHL at +1 SD	0.56 (0.46 to 0.66)	0.050	11.15	<.001	

a FC: face concern.

b SMU: social media usage.

c EHL: eHealth literacy.

d HC: health consciousness.

e SRB: sugar reduction behavior.

Figure 3 demonstrates that when individuals have a higher level of FC, the positive influence of SMU on conformity is stronger (β=.53, 95% CI 0.43-0.62; *P*<.001). Conversely, when individuals have a lower level of FC, the positive impact of SMU on conformity is weaker (β=.35, 95% CI 0.23-0.46; *P*<.001), indicating that FC intensifies the process by which SMU leads to conformity.

**Figure 4.**
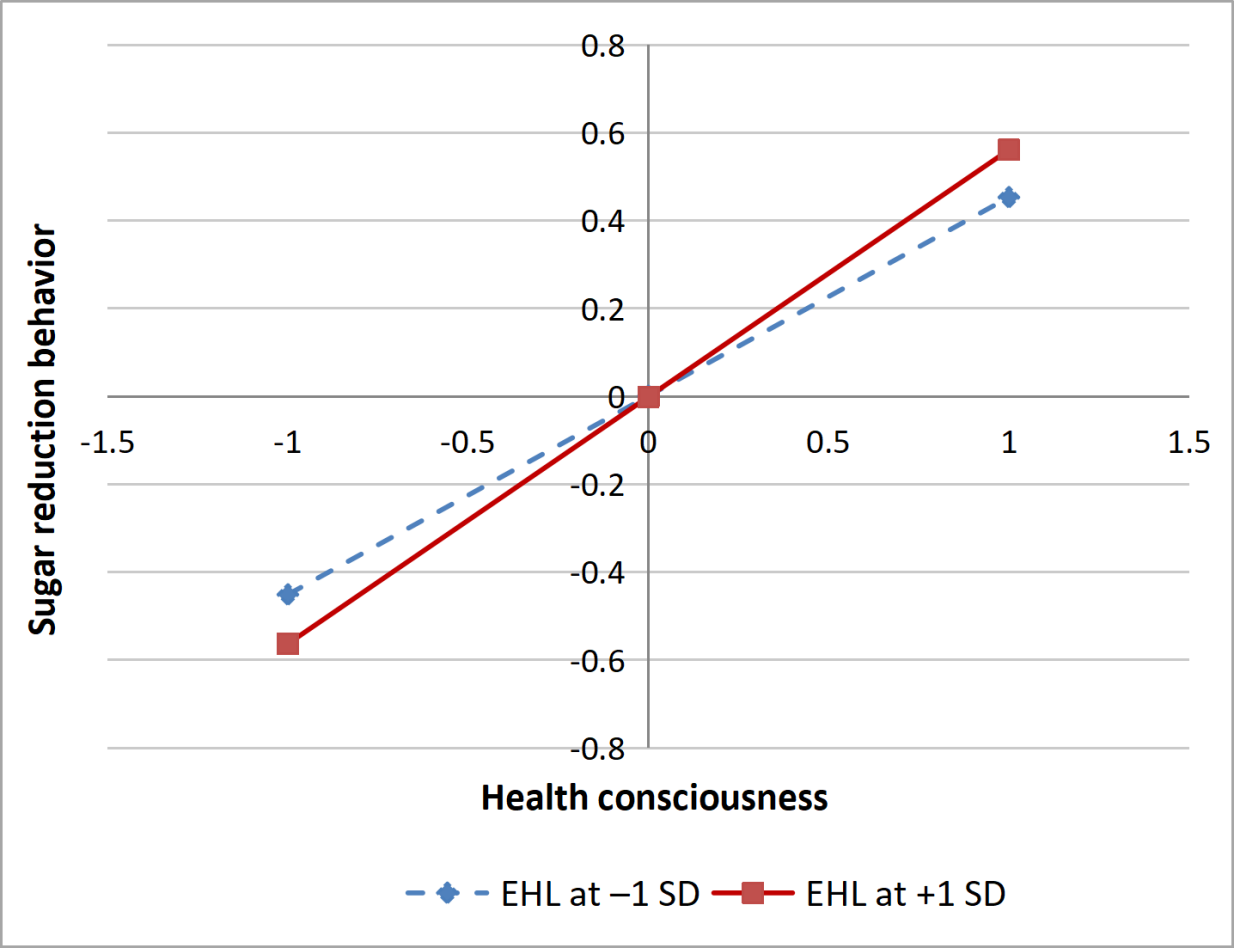
eHealth literacy (EHL) moderating effect diagram.

Figure 4 shows that when individuals possess a higher level of EHL, the effect of HC on SRB is relatively stronger (β=.56, 95% CI 0.46-0.66; *P*<.001). When individuals have a lower level of EHL, the effect of HC on SRB is relatively weaker (β=.45, 95% CI 0.36-0.55; *P*<.001), indicating that EHL can enhance the role of HC in directing individuals toward SRB.

## Discussion

### Principal Findings

This study advances existing research by providing empirical evidence that SMU, HC, and conformity are significant predictors of SRBs among Chinese youth. Notably, HC emerged as the primary mediating pathway between social media engagement and sugar reduction, while conformity played a secondary role. Furthermore, our findings highlight the unique cultural mechanism by which FC positively moderates the influence of social media on conformity and demonstrate that EHL strengthens the translation of HC into actual behavioral change. By integrating FC and EHL into the classic SOR framework, this study offers new theoretical insights into how digital environments and cultural factors interact to shape health behaviors. These results provide a practical foundation for designing culturally tailored interventions to promote healthy dietary habits among youth in collectivistic societies.

### Dual-Pathway Effects of Social Media Use on SRB

For a long time, mass media (television, radio, and newspapers) have been an important tool for health promotion. In recent years, social media has become a key channel for disseminating nutrition and sugar reduction information due to its accessibility and interactive advantages. Based on the results of this study, we verified the 2-path model of social media affecting hypoglycemic behavior: one is the cognitive-led path, where exposure to trustworthy health content can significantly improve health awareness, which is consistent with the health belief model [67]; this shows that even in the collectivist context, adolescents still have the ability of self-directed, belief-based choice. The second is a culturally contextualized conformity path—less sensitive but stable to online social normative pressures, in line with social influence theory [68] and East Asian “relational selves.” The findings suggest that digital health communication should be based on cognitive cues and supplemented with culturally appropriate social norms. While ensuring the credibility and operability of information, peer- and face-related cues should be used prudently, avoiding stigmatization and adverse effects.

Based on this study, social media’s “2-way channel effect” is proposed to construct the “4-in-1” implementation framework further. First, international health agencies or the public sector should establish high-quality digital nutrition content standards (such as information traceability, concise thresholds for free sugars, and actionable alternatives) and coordinate these with structural nutrition policies. Second, public-interest prioritization should be introduced in the governance of media platforms to enhance the visibility of authoritative health information. Transparent sponsorship logos and targeted delivery restrictions on high-sugar content for minor groups should be implemented. Contextual real-time tips are provided in the search stream, such as links to “read sugar labels” micro tutorials. Third, high-frequency, low-load microcontent (goal setting, label reading, and alternative lists) should be deployed along the cognitive path in public health places and school environments. The positive frameworks of peer-led challenges, commitment badges, and face-family well-being (collective benefits) are prudently used in the normative path, and digital and media literacy plus sugar-reduction skills are incorporated into the school-based curriculum, enabling online-offline transformation. Fourth, monitor-assess-iterate: develop institutional indicators, test different cultural frameworks and narratives, and continuously track equity impacts (sex, socioeconomic status, and region). Based on this, iterative optimization should be conducted. Australia’s LiveLighter “Sugary Drinks” campaign promotes risk perception and reduces sugary-drinks consumption through high-frequency, short-range, and strong visual imagery; Singapore’s Nutri-Grade beverage classification policy is synchronized with social media science; and Singapore’s national, the United States’ national “Rethink Your Drink” campaign uses websites, social media toolkits, and school curricula to promote the use of water instead of sugar. Multiple reviews further demonstrate that combining “high-frequency trusted microcontent plus structured support” is methodologically and practically transferable and productive across different cultures and platform contexts.

### Face-Driven Conformity in Digital Health Contexts

Extending the disease-transmission model of conformity of Morsky et al [69], we find a positive association between social media exposure and adolescents’ SRBs, and we differentiate effects by channel: aggregated social media is the strongest predictor, followed by public social media and professional health platforms, whereas official media is not significant. This hierarchy is consistent with a face-concern mechanism operating offline (community reputation and local norms [70]) and online via “digital face”—quantified visibility (likes and comments), traceable reputation archives (interaction histories), and algorithmic reinforcement of majority cues [71]. Aggregators amplify digital-face signals through multisource information aggregation and high visibility; public platforms add diffusion (broad and cross-regional reach) and interpersonal trust (peer and kin pathways); professional sites contribute domain expertise but with narrower networks; and by contrast, official outlets’ single-source, announcement-centric content reduces readability and human relevance for day-to-day nutrition choices, limiting conformity effects.

Culturally, relative to Western individualism that prioritizes personal autonomy and skepticism toward authority [72], Chinese collectivism elevates sensitivity to hierarchy and collective face, strengthening conformity to influencers and salient peer norms [73]. We also observe offline-online synergy: online face presentation evokes offline relational memories, while offline face maintenance makes online behavior instrumentally necessary, jointly steering choices through consistency demands [49]. Credibility and trust are the master pathway: multisource environments (aggregators and public platforms) enable cross-verification and heighten perceived credibility, increasing adoption; single-voice channels (official media) are disadvantaged in competitive attention markets.

Furthermore, it would be wise to minimize the emphasis on indicators of minor vanity and reduce the pressure on “digital faces.” It should draw on face affirmation frameworks (eg, family well-being and collective interests), such as the importance of the “face,” especially in public health and school settings. Cognitive pathways should be targeted with high-frequency, low-burden microcontent. Media literacy and sugar-reduction skills should be incorporated into school curricula, linking online tips to offline choices, and adjusting strategies according to face orientation and exposure frequency. To strengthen public health efforts, governments and global health agencies should provide aggregators with authoritative nutrition guidance, set clear and achievable “sugar reduction” targets, combine communication efforts with structural measures, and select and implement a positive warning or labeling system. This will ensure that cognitive gains are translated into sustained behavioral change. In platform governance, high-sugar promotions for youth should be limited, and real-time tips linked to media content should be incorporated.

This channel-specific, face-informed account integrates historically rooted cultural logic with digitally mediated influence, increasing the model’s explanatory power and policy utility for scalable, context-sensitive sugar-reduction interventions.

### EHL: A Key to Bridging the “Knowledge-Action Gap” in Youth Health

This study systematically reveals the critical role of EHL in transforming health behaviors. Despite the WHO’s clear guidelines on sugar intake, compliance remains suboptimal among youth, primarily due to a widespread perception that they are not at risk for health problems related to sugar consumption [74]. Our findings indicate that even when young individuals possess health awareness and understand the importance of dietary choices, this knowledge does not automatically translate into healthier behaviors, highlighting a significant “knowledge-action gap.” The results emphasize the importance of EHL, which enables individuals to effectively access, understand, and apply health information. It directly influences perceived behavioral control, shapes health beliefs, and strengthens behavioral intentions—critical components for improving sugar-reduction practices.

Empirical evidence from previous studies underscores EHL’s positive impact on health behaviors, particularly among individuals with chronic conditions such as diabetes. In addition, it has been shown to promote community self-care, facilitate the adoption of healthier lifestyles, and reduce health disparities at the community level [75]. This study corroborates and extends these findings, providing robust evidence of EHL’s essential role in promoting low-sugar dietary behaviors among youth. Given the clear gap between health knowledge and behavioral change, this research calls for the integration of EHL enhancement into youth health promotion strategies. These initiatives would not only bridge the knowledge-action gap but also provide actionable solutions for improving dietary habits and promoting long-term health outcomes.

From a global health practice perspective, these findings have significant implications for both global health policy and public health practice. Governments and health organizations should prioritize digital literacy campaigns aimed at enhancing eHealth knowledge among youth, particularly in regions where sugar-related diseases are on the rise. These initiatives could include school-based educational programs, digital health toolkits, and public health campaigns designed to engage youth with accurate, accessible health information. Moreover, public health campaigns should focus on improving the digital accessibility of health information, ensuring that youth can easily engage with and understand health messages on social media and other digital platforms. Promoting EHL should be considered a core pillar of global public health efforts to reduce sugar consumption and mitigate associated health conditions, especially in light of the increasing prevalence of chronic diseases among youth worldwide.

### Platform-Specific Considerations and Cross-Platform Generalizability

While this study conceptualizes SMU as a holistic construct to reveal cross-platform cultural mechanisms, we acknowledge that platform-specific architectures (eg, WeChat’s private networks vs Douyin’s [ByteDance Ltd] algorithmic feeds) may introduce behavioral nuances. Crucially, our core moderator—FC—exerted a consistent influence across platforms (β=.089; *P*=.02), suggesting it operates through enduring social norms rather than platform-specific design cues [76]. This cross-platform consistency is substantiated by 3 convergent pieces of evidence. First, cultural moderators show invariant effects (β=.07‐0.12; *P*<.05) in collective societies across studies [77]; second, adolescents exhibit norm-driven consistency regardless of platform type, evidenced by alcohol-risk cognition correlations (*r*=0.71; Facebook-MySpace [Tom Anderson and Chris DeWolfe] [78]); and third, 87% of adolescents maintain stable health behaviors across platforms globally [79]. Collectively, this body of evidence validates our approach, given participants’ multiplatform engagement, empirically anchoring in Chinese participants’ multiplatform ecology: 95.76% used WeChat, 65.57% used Douyin, and 35.91% used Weibo (Sina Corporation), with over 87% actively engaging on ≥3 platforms simultaneously [80]. These results also underscore the external validity and real-world relevance of our sample, as participants’ engagement patterns mirror contemporary youth behavior in China’s dynamic digital landscape.

Our findings indicate that cross-platform consistency in adolescent behavior should be treated as a design constraint for digital health communication. Because young people routinely engage with multiple platforms, interventions ought to be platform-sensitive (tailored to architectural features such as private-network diffusion vs algorithmic feeds) while centering shared cross-platform mechanisms, notably FC as a cultural moderator. In collectivist Chinese contexts, this implies prioritizing face-affirming frames (family well-being and collective benefit) and responsible social proof over purely informational appeals, coupled with safeguards against excessive normative pressure that can induce stigma or reactance.

International precedents support this multiplatform orchestration. The United Kingdom’s Change4Life (“Smart Swaps” and “Sugar Smart”) coordinated broadcast, app, and social media assets to promote lower-sugar substitutions, reporting measurable shifts in purchasing and nutrition knowledge. In the United States, “Rethink Your Drink” leveraged websites and school-based social content to normalize water substitution and lower-sugar swaps, with improvements in curriculum-linked knowledge and self-reported intake. Adapting this template to China’s digital ecology—while explicitly modeling FC as a cross-platform moderator—offers a theoretically coherent and operationally scalable pathway for youth sugar-reduction efforts.

### Limitations

Key limitations necessitate cautious interpretation of the findings, and methodological refinements for future research are therefore recommended.

First, reliance on a single web survey and self-administered questionnaires may introduce common methodological biases that limit the generalizability of causal inference and influence results. Using a single data collection method limits the ability to draw clear causal conclusions and may affect the applicability of findings to different groups. While safeguards such as poststratification weighting have been implemented to better represent the national youth population, the underrepresentation of rural or low-literacy groups remains a constraint. This discrepancy affects the generalizability of our conclusions and limits the interpretation of findings from different population studies. To address this issue, future research should explore mixed-mode sampling, adopt a staged approach, and incorporate offline or community recruitment strategies to increase the inclusiveness and robustness of survey results. While social desirability bias is a common challenge for online self-report methods, our procedure aims to minimize its impact, and the results are consistent with previous studies. However, research methods should be refined, especially through integrated mixed methods, to improve the validity and reliability of future studies and address the limitations of relying solely on online surveys.

Second, the cross-sectional design of this study represents a significant limitation, as it restricts the ability to draw causal conclusions. While we attempted to mitigate this limitation by confirming predictive validity (partial least squares structural equation modeling: Q²=0.113>0.05) and validating the moderating role of EHL in line with Bandura’s temporal precedence theory [81], these methods fall short of establishing clear temporal precedence between the variables. Without establishing temporal causality, the conclusions drawn are limited to associations rather than causal relationships. Longitudinal tracking is, therefore, essential for future research to fully address causal relationships. To this end, our forthcoming Ecological Momentary Assessment study is designed to track the real-time interplay between HC, conformity, and SRBs, allowing for a more granular understanding of these dynamic causal relationships over time. This will provide further insight into the causal mechanisms driving health behavior changes.

Third, although our adapted SRB scale demonstrated strong psychometric properties, it does not fully capture culturally embedded behaviors, such as peer influence during group dining experiences. A key limitation of our scale is its reliance on self-reporting, which may introduce response biases, particularly social desirability bias, as participants may tend to report behaviors they perceive as socially acceptable. In addition, while the scale was adapted for the Chinese context, it may still be influenced by cross-cultural issues, as cultural norms and expectations differ across societies, particularly in collectivist cultures such as China. Future research should refine and validate the measurement tools by integrating physiological monitoring of real-time behaviors and developing cross-culturally validated metrics that better reflect local norms and social contexts. Such improvements will enhance the scale’s ability to measure culture-specific behaviors accurately and ensure the validity and reliability of behavioral assessments in diverse cultural settings.

Furthermore, while our findings indicate significant cross-platform effects, the study does not fully explore how different platforms, user motivations, and engagement styles might influence SRBs differently. In addition, the psychological mechanisms underlying conformity remain inadequately understood, highlighting the need for further investigation. Future studies should adopt platform-specific follow-up and mixed methods designs to unravel these complex mechanisms. This will allow for developing more targeted interventions that effectively promote SRBs in digital environments.

### Conclusions

This study examines social media’s role in shaping SRBs among Chinese youth, finding that it promotes such behaviors mainly by increasing health awareness and secondarily by influencing conformity due to FCs. EHL positively moderates these effects. The marginal contribution of the research lies in its novel application of the SOR framework to investigate the mechanisms of social media’s impact on SRBs among youth, emphasizing the interplay between conformity and HC. By combining insights from communication studies and psychology, it reveals the regulatory effects of FC and EHL in Eastern cultures, thereby enhancing our understanding of the theoretical mechanisms in public health management and health communication, and offering a systematic and culture-specific exploration of health communication principles and their cultural variations, providing more diverse and in-depth views to the global health communication discourse.

Leveraging the culturally adaptive SOR framework, digital health interventions can transcend simple information dissemination to empower users cognitively, fostering HC and EHL that translate health stimuli into sustained SRB. Operationally, this means embedding culturally tailored strategies—like peer education, social media role models, and community-based challenges—within health education initiatives to align with regional values, particularly Eastern collectivist norms and “face” dynamics that foster social accountability. The model’s scalability further enables coordinated efforts across education, health, and media sectors, supporting algorithmic and monitoring systems that responsibly guide and supervise health information. Crucially, ensuring algorithmic and policy ethics—by regulating targeted promotion of unhealthy products and embedding cultural value assessments in intervention design—is vital for achieving equitable and effective outcomes. Together, these approaches comprise a “digital-cultural behavior framework,” shifting the paradigm from generic awareness campaigns to context-sensitive, cognitively empowering, and culturally grounded digital health solutions. This framework provides a transferable blueprint for deploying technology-driven public health strategies tailored to the diverse sociocultural realities of today’s youth worldwide.

## Supplementary material

10.2196/68180 Multimedia Appendix 1 The questionnaire used in the survey.

10.2196/68180 Multimedia Appendix 2 Demographic statistics of Chinese youth and poststratification-weighted structural equation model (SEM) results.

10.2196/68180 Multimedia Appendix 3 Reliability and validity metrics and model fit indices.

## Abbreviations

EHL: eHealth literacy
FC: face concern
HC: health consciousness
HCS: Health Consciousness Scale
SEM: structural equation model
SKI-N: Informational and Normative Conformity Scale
SMU: social media usage
SOR: stimulus-organism-response
SRB: sugar reduction behavior
WHO: World Health Organization
