# Supplementary material for: Social Media, Health Consciousness, and Cultural Influences on Sugar Reduction Behaviors in Chinese Youth: Extending the Stimulus-Organism-Response Model
Source: J Med Internet Res. 2025 Dec 19;27:e68180. doi: 10.2196/68180 (PMC12716413; doi:10.2196/68180)
Supplement: Multimedia Appendix 1 [file jmir-v27-e68180-s001.doc]

A Survey on Sugar Reduction Behavior of Chinese Youth

We are from the School of Journalism and Communication, currently conducting a research study on the low-sugar dietary intentions and behaviors of Chinese youth. This questionnaire aims to explore the status of low-sugar dietary practices among young people in the age of social media. Your insights and experiences are invaluable to us, and we sincerely appreciate your participation! Please rest assured that your responses will remain anonymous and confidential, in accordance with the National Statistical Law. Feel free to answer openly and honestly.

School of Journalism and Communication

Huaqiao University

June 2024

**1. Your gender is:**

1) Male

2) Female

**2. Your age is:**

1) 15-18 years old

2) 18-24 years old

3) 24-30 years old

4) 30-35 years old

4) 35 years old and above

**3. What type of specialty is your educational background closer to?**

1) Liberal arts

2) Science and engineering

3) None of above

**4. What is your highest level of education**

1) Below junior high school level

2) High school/secondary vocational education

3) Junior college

4) Undergraduate

5) Postgraduate and above

**5. Please read the following sentences and choose the number that most fits your idea. The bigger the number is, the more you agree with it.** (1 =totally disagree, 5 =totally agree)

| **Sugar Control Behavior** | totally disagree totally agree | | | | |
| --- | --- | --- | --- | --- | --- |
| 1)I choose low-sugar foods and beverages. | 1 | 2 | 3 | 4 | 5 |
| 2) I deliberately reduce high-sugar diets and sugary foods. | 1 | 2 | 3 | 4 | 5 |
| 3) I pay special attention to the sugar content labels on food and beverage packaging. | 1 | 2 | 3 | 4 | 5 |

***6. Please read the following sentences and choose the number that most fits your idea. The bigger the number is, the more you agree with it.*** *(1 =totally disagree, 5 =totally agree)*

|  | totally disagree totally agree | | | | |
| --- | --- | --- | --- | --- | --- |
| 1) In order to get likes and favorable comments on social media, I sometimes reserve or do not fully express my true thoughts. | 1 | 2 | 3 | 4 | 5 |
| 2) In order to gain acceptance from my friends, I sometimes accept their points of view without thinking deeply about them. | 1 | 2 | 3 | 4 | 5 |
| 3) On social media, the opinions of people I respect are important to me, and I tend to form my own opinions under the influence of their opinions. | 1 | 2 | 3 | 4 | 5 |
| 4) To avoid controversial conflicts on social media, I sometimes act against my own views or principles. | 1 | 2 | 3 | 4 | 5 |
| 5) I believe it is important to align with the majority on social media, which often leads me to adopt the viewpoints of the majority. | 1 | 2 | 3 | 4 | 5 |
| 6) Worrying about posting inappropriate content, I sometimes choose to follow the opinions or behaviors of others on social media. | 1 | 2 | 3 | 4 | 5 |
| 7) When uncertain of my own opinions, I tend to refer more to the opinions of others on social media. | 1 | 2 | 3 | 4 | 5 |
| 8) Faced with complex topics on social media, I prefer to follow the majority opinion to reduce the risk of making bad decisions. | 1 | 2 | 3 | 4 | 5 |
| 9) When in a difficult situation, I usually act in accordance with the opinion of the majority, believing that they know better how to deal with it. | 1 | 2 | 3 | 4 | 5 |
| 10) I care about others' praise and criticism. | 1 | 2 | 3 | 4 | 5 |
| 11) I care about others' attitudes towards me | 1 | 2 | 3 | 4 | 5 |
| 12) I hate being looked down upon. | 1 | 2 | 3 | 4 | 5 |
| 13) I get very angry if people are rude to me. | 1 | 2 | 3 | 4 | 5 |
| 14) I am very pleased to receive respect. | 1 | 2 | 3 | 4 | 5 |
| 15) I would feel very frustrated if I were publicly criticized. | 1 | 2 | 3 | 4 | 5 |

**7. How many friends do you have on various social media? (e.g. WeChat, QQ, Weibo, TikTok, RED, Bilibili, etc.)**

1)10 or less

2)11-50

3)51-100

4)101-150

5)151-200

6)201-250

7)251-300

8)301-400

9)400 or more

**8. On average, how much time have you spent daily on different types of social media over the past week?**

1)Less than 30 minutes

2)30-60 minutes

3)1-2 hours

4)2-4 hours

5)4-6 hours

6)6-8 hours

7)8-12 hours

8)12 hours or more

*9. Read the following sentences and choose the number that most fits your idea. The bigger the number is, the more you agree with it. (1 =totally disagree, 5 =totally agree)*

|  | totally disagree totally agree | | | | |
| --- | --- | --- | --- | --- | --- |
| 1) Using social media is a part of my life | 1 | 2 | 3 | 4 | 5 |
| 2) I am proud to use social media. | 1 | 2 | 3 | 4 | 5 |
| 3) I spend most of my time on social media. | 1 | 2 | 3 | 4 | 5 |
| 4) When I don't use social media, I feel a sense of loneliness. | 1 | 2 | 3 | 4 | 5 |
| 5) I feel a strong connection to the social media community. | 1 | 2 | 3 | 4 | 5 |
| 6) Turning off social media would be hard and regrettable for me. | 1 | 2 | 3 | 4 | 5 |

***10. This part is about eHealth literacy. Please read the following sentences and choose the number that most fits your idea. The bigger the number is, the more you agree with it.*** *(1 =totally disagree, 5 =totally agree)*

|  | totally disagree totally agree | | | | |
| --- | --- | --- | --- | --- | --- |
| 1) I am aware of the types of health information available online. | 1 | 2 | 3 | 4 | 5 |
| 2) I know how to find useful health information on the internet. | 1 | 2 | 3 | 4 | 5 |
| 1. I know where to find helpful health information online. | 1 | 2 | 3 | 4 | 5 |
| 4) I know how to use the Internet to answer my questions about health. | 1 | 2 | 3 | 4 | 5 |
| 5) I know how to use the internet to address my health concerns. | 1 | 2 | 3 | 4 | 5 |
| 6) I have the skills to evaluate the health resources I find on the Internet. | 1 | 2 | 3 | 4 | 5 |
| 7) I can tell high-quality health resources from low-quality health resources on the Internet. | 1 | 2 | 3 | 4 | 5 |
| 8) I feel confident in using information from the Internet to make health decisions. | 1 | 2 | 3 | 4 | 5 |

*11. This part is about health consciousness. Please read the following sentences and choose the number that most fits your idea. The bigger the number is, the more you agree with it. (1 =totally disagree, 5 =totally agree)*

|  | totally disagree totally agree | | | | |
| --- | --- | --- | --- | --- | --- |
| 1. I'm alert to changes in my health | 1 | 2 | 3 | 4 | 5 |
| 1. I reflect about my health a lot | 1 | 2 | 3 | 4 | 5 |
| 1. I regularly assess my health status to identify potential issues. | 1 | 2 | 3 | 4 | 5 |
| 1. I am highly concerned about maintaining good health. | 1 | 2 | 3 | 4 | 5 |
| 1. I pay attention to my body's signals regarding my health. | 1 | 2 | 3 | 4 | 5 |
| 1. I routinely check my health status to ensure well-being. | 1 | 2 | 3 | 4 | 5 |
| 1. I'm very involved with my health | 1 | 2 | 3 | 4 | 5 |
| 1. During work hours, I keep a mindful awareness of my health status. | 1 | 2 | 3 | 4 | 5 |
| 1. I'm aware of the state of my health as I go through the day | 1 | 2 | 3 | 4 | 5 |

**12. How long have you been using social media platforms (WeChat, Weibo, TikTok, Toutiao, Bilibili, etc.)?**

1）3 years or less

2）3-5 years

3）5-10 years

4）10 years and above

1. **Please determine if the statement is true: "Carbohydrates are an essential nutrient for the human body."**

1）True

2）False

3）Not Sure

**14. What is your approximate weight? Please select the closest weight range from the options below:**

1）Below 40 kilograms

2）40-49 kilograms

3）50-59 kilograms

4）60-69 kilograms

5）70-79 kilograms

6）80-89 kilograms

7）90 kilograms and above

**15. Which of the following ranges does your height fall into?**

1）Below 140 centimeters

2）140-149 centimeters

3）150-159 centimeters

4）160-169 centimeters

5）170-179 cm

6）180-189 cm

7）190 cm and above

**16. Where does your family live？**

1）Rural

2）Town

1. **Please determine if the statement is true: "Sugar is a tasteless substance."**

1）True

2）False

3）Not Sure

1. **Which of the following Chinese social media platforms do you use regularly? (Please tick ALL that apply.)**
   1）WeChat
   2）Weibo
   3）Douyin/TikTok
   4）Xiaohongshu/Rednote
   5）QQ
   6）Bilibili
   7）Zhihu
   8）Baidu Tieba

9）Zhihu
10）Other (please specify): _____________

**19. Please rate your health over the past six months using the following options:**

1）Very good

2）good

3）Average

4）Poor

5）Very poor

**Thanks for your answers and support!**
