# Supplementary material for: Social Media, Health Consciousness, and Cultural Influences on Sugar Reduction Behaviors in Chinese Youth: Extending the Stimulus-Organism-Response Model
Source: J Med Internet Res. 2025 Dec 19;27:e68180. doi: 10.2196/68180 (PMC12716413; doi:10.2196/68180)
Supplement: Multimedia Appendix 2 [file jmir-v27-e68180-s002.docx]

**Table A1**. Demographic profile of Chinese youth (postweight values, ages 15-35^a^).

| Category​​ | **​**​Data​​ | **​**Source​​ |
| --- | --- | --- |
| Age Distribution​​ | 15-35 years: 27.2% of the total population | National Bureau of Statistics of China. (2021). Seventh national population census. http://www.stats.gov.cn/english/PressRelease/202105/t20210510_1817185.html |
|  | • 15-24 years: 15.89% |  |
|  | • 25-35 years: 11.31% |  |
| Gender Ratio​​ | Sex ratio: 105.4 (males per 100 females) | National Bureau of Statistics of China. (2021). Seventh national population census. |
|  | • Male: 52.1% |  |
|  | • Female: 47.9% |  |
| Education Level​​ | Below junior high school: 40.3% | (i) National Bureau of Statistics of China. (2024). China Statistical Yearbook 2024. Retrieved from  <https://www.stats.gov.cn/sj/ndsj/2024/indexch.htm>  (ii) Ministry of Education of the People's Republic of China. (2023). China Educational Statistical Yearbook. Retrieved from <http://www.moe.gov.cn/jyb_sjzl/moe_560/2023/> |
|  | High school/secondary vocational: 28.8% |  |
|  | Junior college: 14.9% |  |
|  | Undergraduate and above: 16.0% |  |
| Residence Type​​ | Urbanization rate: 66.16% | National Bureau of Statistics of China. (2024). China Statistical Yearbook 2024. |
|  | • Urban residents: 63.89% |  |
|  | • Rural residents: 36.11% |  |

^a^ Data restricted to 15-35 cohort using: $\text{P}_{\text{adj}}\text{=}\text{P}_{\text{total}}\text{×}\frac{\text{N}_{\text{15-35}}}{\text{N}_{\text{15+}}}$

**Table A2.** Poststratification-weighted SEM results^a.^

| Path | Unweighted β | Weighted β | Δβ | Δ% | p-value (U) | p-value (W) | CI Overlap |
| --- | --- | --- | --- | --- | --- | --- | --- |
| SMU → SRB | 0.082 | 0.081 | -0.001 | -1.20% | 0.04 | 0.042 | Yes |
| SMU →HC | 0.353 | 0.348 | -0.005 | -1.40% | <0.001 | <0.001 | Yes |
| HC→ SRB | 0.498 | 0.491 | -0.007 | -1.40% | <0.001 | <0.001 | Yes |
| SMU →CFM | 0.508 | 0.503 | -0.005 | -1.00% | <0.001 | <0.001 | Yes |
| CFM → SRB | 0.139 | 0.143 | 0.004 | 2.90% | <0.001 | <0.001 | Yes |
| SMU × FC→ CFM | 0.089 | 0.092 | 0.003 | 3.40% | 0.02 | 0.018 | Yes |
| HC× EHL →SRB | 0.055 | 0.054 | -0.001 | -1.80% | 0.04 | 0.042 | Yes |

^a^After weighting, all path coefficients shifted by <0.02 and their 95 % CIs overlapped; substantive findings remain stable.
