# Supplementary material for: Social Media, Health Consciousness, and Cultural Influences on Sugar Reduction Behaviors in Chinese Youth: Extending the Stimulus-Organism-Response Model
Source: J Med Internet Res. 2025 Dec 19;27:e68180. doi: 10.2196/68180 (PMC12716413; doi:10.2196/68180)
Supplement: Multimedia Appendix 3 [file jmir-v27-e68180-s003.docx]

**Complete reliability and validity metrics**

**Table B1.** Validation results of the sugar reduction behavior scale adapted from the HPLP-II.

| Validation Step (SRB) | Statistic / Index | Result / Threshold | Notes |
| --- | --- | --- | --- |
| 1. Content Validity | Expert panel (n = 8) |  |  |
| Item-CVI (I-CVI) | 0.89 | > 0.80 | 4-point Likert |
| Scale-CVI (S-CVI/Ave) | 0.92 | > 0.80 |  |
| 2. Sampling Adequacy | Kaiser-Meyer-Olkin (KMO) | 0.812 | > 0.70 |
| 3. Sphericity Test | Bartlett’s χ² | 287.35 | p < .001 |
| 4. Exploratory Factor Analysis (EFA) 30 % subsample (n = 265) |  |  |  |
| Factor structure | Unidimensional | – | Eigenvalue > 1 |
| Explained Variance | 68.30% | > 50 % |  |
| Factor Loadings Range | 0.65 – 0.79 | All > 0.50 |  |
| 5. Confirmatory Factor Analysis (CFA) 70 % hold-out (n = 618) |  |  |  |
| Model Fit | χ²/df = 2.18 | < 3.00 |  |
|  | CFI = 0.948 | > 0.95 |  |
|  | RMSEA = 0.068 | < 0.08 |  |
| Standardized Loadings | 0.71 – 0.79 | All p < .001 |  |
| 6. Reliability | Cronbach’s α | 0.85 | > 0.70 |
| 7. Convergent Validity | Average Variance Extracted (AVE) | 0.81 | > 0.50 |
|  | Composite Reliability (CR) | 0.85 | > 0.70 |
| 8. Criterion Validity | Correlation with self-reported sugar intake | r = –0.41, p < .001 | Expected negative |

**Table B2.** Correlation analysis and discriminant validity (Fornell and Larcker Criterion).

|  | AVE | CFM | EHL | FC | HC | SRB | SMU |
| --- | --- | --- | --- | --- | --- | --- | --- |
| CFM | .56 | **.74^a^** |  |  |  |  |  |
| EHL | .55 | .18^b^ | **.74** |  |  |  |  |
| FC | .51 | .32 | .35 | **.72** |  |  |  |
| HC | .57 | .23 | .64 | .32 | **.75** |  |  |
| SRB | .81 | .30 | .37 | .14 | .56 | **.90** |  |
| SMU | .51 | .51 | .37 | .39 | .38 | .34 | **.71** |

^a^The bold values indicate the square root of AVE

^b^Pearson correlation in the crosstabulation of variables

**Table B3.** Common method bias check (VIF of latent variables).

| Endogenous Variable | Predictor Variable | VIF |
| --- | --- | --- |
| HC | SMU | 1.35 |
| CFM | FC | 1.23 |
| CFM | SMU | 1.19 |
| SRB | HC | 1.78 |
| SRB | CFM | 1.35 |
| SRB | SMU | 1.54 |

**Model fit indices and comparisons**

**Table C1.** Criteria for alternative models**.**

|  | Critieria | A_model1  (sequential) | A_model2  (Parallel) | A_model3  (CFM→HC) | model4  (proposed) |
| --- | --- | --- | --- | --- | --- |
| PLS-SEM  based | *R*^2^ | .336 | .327 | .320 | .327 |
|  | adjusted *R*^2^ | .335 | .325 | .318 | .325 |
| incremental validity | AIC | -356.57 | -342.68 | -344.68 | -342.68 |
|  | AICu | -353.56 | -338.67 | -341.67 | -338.67 |
|  | FPE | .668 | .678 | .677 | .678 |
| incremental consistency | BIC | -342.22 | -323.54 | -330.33 | -323.54 |
|  | GM | 890.56 | 909.13 | 893.27 | 909.13 |
|  | HQ | -351.08 | -335.36 | -339.19 | -335.36 |
| PLS predict | Q²predict | .093 | .113 | .108 | .113 |
|  | RMSE | .955 | .944 | .947 | .944 |
|  | MAE | .762 | .735 | .748 | .735 |
| PLS  model fit | SRMR | .089 | .073 | .073 | .072 |
|  | Chi-square | 2310.36 | 1774.38 | 1799.62 | 1794.21 |
|  | NFI | .888 | .892 | .890 | .890 |

**Table C2.** CVPAT results for alternative models**.**

| Comparison | CVPAT results | Average Losses | | |  |
| --- | --- | --- | --- | --- | --- |
|  |  | EM | AM | EM-AM^b^ | *P* value^c^ |
| 1 | EM(Model4) and AM(Model1)^a^ | 1.09 | 1.092 | -0.002 | .32 |
| 2 | EM(Model4) and AM(Model2) | 0.945 | 0.945 | 0 | .04 |
| 3 | EM(Model4) and AM(Model3) | 0.945 | 0.946 | -0.001 | .47 |

^a^EM refers to the established model; AM denotes the alternative model. The null hypothesis posits equivalent predictive capabilities, while the alternative hypothesis suggests that AM (fourth column) outperforms EM (third column) in predictive accuracy.

^b^A negative difference in mean loss values between EM and AM indicates that EM has a lower mean loss, thus being preferable. A negative mean loss difference implies superior predictive ability for EM.

^c^*P* values are derived from the PLSpredict/CVPAT module in SmartPLS 4.0 software, with 10 folds and 10 repetitions, utilizing a fixed seed.

**Table C3**. Control-variable estimates in the PLS-SEM direct-effects model.

| Control variables | β (95% CI) | SE^b^ | t^c^ | *P* value |
| --- | --- | --- | --- | --- |
| Gender | –.06 (–0.19 to 0.07) | 0.063 | 0.98 | .33 |
| Major | –.23 (–0.35 to –0.11) | 0.063 | 3.67 | <.001 |
| Education | .028 (–0.02 to 0.08) | 0.027 | 1.02 | .31 |
| BMI | .07 (0.01-0.13) | 0.030 | 2.35 | .02 |
| Health | –.08 (–0.14 to –0.02) | 0.032 | 2.42 | .02 |
